# Z-score values of left ventricular dimensions in adolescent elite male soccer players

**DOI:** 10.1007/s00431-020-03741-1

**Published:** 2020-07-24

**Authors:** Stephan Gerling, Tobias Pollinger, Holger Michel, Markus-Johann Dechant, Michael Melter, Werner Krutsch

**Affiliations:** 1 Department of Pediatrics, University Children’s Hospital Regensburg (KUNO), Campus St. Hedwig, Regensburg, Germany; 2 Department of Visceral Surgery, Hospital Barmherzige Brüder, Regensburg, Germany; 3 Department of Traumatology, University Hospital Regensburg, Regensburg, Germany

**Keywords:** Sudden cardiac death, Echocardiography, Soccer, Athletes, Adolescent, Z-scores

## Abstract

Recent studies showed contrasting findings in morphological changes due to competitive soccer in adolescent players (SP). We present a prospective study in 315 consecutive adolescent (10–14 years) male elite SP and 53 healthy matched active controls (CON). All participants underwent a complete transthoracic two-dimensional echocardiography (TTE). The mean age in SP was 12.8 ± 0.65 years compared to 12.6 ± 0.8 years in CON. For all left ventricular (LV) dimensions, mean Z-score values were higher in SP. There was a significant Z-score increase in interventricular septum diastolic diameter (2.47z vs. 1.62z, *p* < 0.05), left ventricular posterior wall diastolic and systolic diameter (1.15z vs. 0.47z, *p* < 0.05 and 1.05z vs. − 0.4z, *p* < 0.05). Athletes had significant greater LV mass indexed for BSA (94 ± 12 g/m^2^ vs. 81 ± 13 g/m^2^, *p* < 0.05). There was no significant difference in LV function or diameters.

*Conclusion*: Our findings suggest that elite soccer training in adolescent male is a type of sport predominantly related to cardiac resistance remodeling. Adolescent SP may develop supernormal left ventricular wall dimensions (+ 2.0 to + 2.5z). If in SP Z-scores, any LV dimension above + 2.5 is measured, primary or secondary cardiomyopathies should be excluded.

**Table:** 

**What is Known:** *• Morphological cardiac adaptation in response to exercise depends on the type, duration, and intensity of training.* *• Morphological and functional changes due to competitive sports (athlete’s heart) occur even in pre-adolescent athletes.*	
**What is New:** *• Our findings point out that German elite soccer training in adolescent male (10–14 years of age) is a type of sport predominantly related to cardiac resistance remodeling.* *• If in an adolescent competitive soccer player any LV dimension Z-score value above + 2.5 is measured, a primary or secondary cardiomyopathy should be excluded.*

**What is Known:**

*• Morphological cardiac adaptation in response to exercise depends on the type, duration, and intensity of training.*

*• Morphological and functional changes due to competitive sports (athlete’s heart) occur even in pre-adolescent athletes.*

**What is New:**

*• Our findings point out that German elite soccer training in adolescent male (10–14 years of age) is a type of sport predominantly related to cardiac resistance remodeling.*

*• If in an adolescent competitive soccer player any LV dimension Z-score value above + 2.5 is measured, a primary or secondary cardiomyopathy should be excluded.*

## Introduction

The Association for European Pediatric Cardiology recommends that cardiovascular screening should be performed on athletes when they start competitive activity. [1] Nowadays in European soccer, competitive sports starts at an age of 12–13 years. Adolescent male elite soccer players (SP) train between 8 and 12 h and play one match per week. In this regard, the cardiovascular system has to increase its work significantly, compared to moderate intensity levels. It is well known that in adolescent athletes, intense and regular exercise does affect size, dimensions, or mass of the left ventricle (LV). [2]

Morphological cardiac adaptation in response to sport depends on the type, duration, and intensity of training. Endurance exercise (e.g., cycling or running) results in an increased cardiac output leading to increased volume load and consecutively to an eccentric LV hypertrophy. Strength exercise (e.g., weightlifting) is characterized by an increased peripheral vascular resistance, which results in increased LV after load and therefore to a concentric LV hypertrophy. [3] There are also overlapped sports combining endurance and strength hemodynamic conditions such as soccer. [4] German elite soccer training in early adolescence focus on ball control exercises, jumps, and run-ups with lots of repetitions to improve motion automation with less attention to endurance training.

## Aims

The aim of our prospective study was to measure left cardiac dimensions and function in elite non-symptomatic male adolescent SP and healthy matched controls using conventional TTE.

## Methods

### Study population

This prospective study was performed over 2 years at a tertiary children’s university hospital. A total of 359 consecutive adolescent male elite SP of the Bavarian Football Association participated in our study. As controls (CON), we selected 53 age- and sex-matched inpatients from the pediatric surgery department of our hospital.

Inclusion criteria were male sex, age 10–14 years, BMI 13–25 kg/m^2^, no history of cardiovascular disease, Caucasian ethnicity, and field player. Two participants with a minor cardiovascular disease and 42 goalkeepers had to be excluded because of different training load. The SP training profiles were as follows: training load of 8.5 ± 3.5 h per week, one competitive match per week, 11 ± 1 months per year training, and 3.5 ± 1.5 years of training. CON were all recreationally active but were not taking part in any regular training scheme. All participants underwent a standardized cardiovascular screening protocol with a medical history, a physical examination, a 12-lead resting electrocardiogram, and a TTE at rest.

The study is in compliance with the Declaration of Helsinki and was approved by the institutional review board of the University of Regensburg (15-101-0079). Informed consent was obtained of all children and/or their legal guardians.

### Echocardiography

Echocardiography (TTE) was performed using a portable cardiovascular ultrasound system (Vivid i, GE Healthcare) with a 6S-RS (2.7–8.0 MHz) or a 3S-RS (1.5–3.6 MHz) transducer. The recent guideline for chamber quantification from the American Society of Echocardiography and the European Association of Cardiovascular Imaging were followed for echocardiographic measurements. [5] All subjects were examined in supine or left/right lateral decubitus position by well-experienced pediatric cardiologists (SG, HM). End-diastolic dimensions and wall thicknesses were calculated as the average of three successive cardiac cycles. LVM was estimated using following equation:

$$ \mathrm{LV}\ \mathrm{mass}\ \left(\mathrm{g}\right)=0.8\left\{1.04\left[\left({\left[\mathrm{LVEDD}+\mathrm{IVSd}+\mathrm{PWd}\right]}^3-{\mathrm{LVEDD}}^3\right)\right]\right\}+0.6 $$

All studies were digitally stored for off-line analysis, retrospective re-evaluation, and follow-up examinations.

### Statistical analysis

Normality of data was assessed using Shapiro-Wilk. For normally distributed data, a Student’s independent *t* test was used. The body surface area (BSA) was calculated with Haycock’s formula. [6] In TTE especially in children and adolescents, it is useful to report quantitative measures within the context of BSA appropriate norms (e.g., Z-score values) in addition to reporting the absolute values. The Z-score is a classification system that provides the standard deviation of a value compared to a normally distributed population and allows comparison and standardization of measurements. [7] Statistical significance was defined as *p* < 0.05. All analyses were conducted using IBM SPSS Statistics, version 25.

## Results

A total of 315 SP and 53 CON were eligible for data interpretation. The mean age in SP was 12.8 ± 0.65 years compared to 12.6 ± 0.8 years in CON. SP had a slightly lower mean body mass index (17.8 ± 1.7 vs. 18.6 ± 2.7) and body surface area (1.38 ± 0.17 vs. 1.43 ± 0.23 m^2^). Both groups had regular mean systolic and mean diastolic blood pressure values at rest. Mean values (mm) for LV dimensions (SP vs. CON): IVSD 8.9 vs. 7.92 mm, IVSS 12.96 vs. 12.32 mm, LVPWD 8.38 vs. 7.63 mm, LVPWS 12.54 vs. 11.50 mm, LVIDD 44.90 vs. 44.42 mm, LVIDS 28.38 vs. 28.09 mm. For all LV dimensions, mean Z-scores were higher in SP than in CON. There was a significant increase in IVSD (2.47z vs. 1.62z, *p* < 0.05), LVPWD (1.15z vs. 0.47z, *p* < 0.05), and LVPWS (1.05z vs. − 0.4z, *p* < 0.05). The mean Z-score of both the diastolic and the systolic diameter of the interventricular septum was above 2.0 (IVSD 2.47z, *p* < 0.05; IVSS 2.23z, *p* > 0.05) in SP. A small subgroup of SP did develop supernormal LV wall Z-score values, but did not exceed + 2.5z. Athletes had significant greater LV mass indexed for BSA (94 ± 12 g/m^2^ vs. 81 ± 13 g/m^2^, *p* < 0.05). There was no significant difference in conventional LV function (EF, LVFS) and LV systolic or diastolic internal diameter (Table 1).

**Table 1:** Demographic characteristics, left ventricular dimensions, and Z-scores

	SP (*n* = 315)	CON (*n* = 53)	*p* values
Age (years)	12.8 (± 0.65)	12.6 (± 0.8)	*p* > 0.05
BMI (kg/m^2^)	17.8 (± 1.7)	18.6 (± 2.7)	*p* > 0.05
BSA (m^2^)	1.38 (± 0.17)	1.43 (± 0.23)	*p* > 0.05
BP sys (mmHg)	112 (± 9)	105 (± 22)	*p* > 0.05
BP dia (mmHg)	60 (± 7)	62 (± 14)	*p* > 0.05
LVMi	*94 g/m*^*2*^ (± 12)	81 g/m^2^(± 13)	*p < 0.05*
LVFS (%)	37 (± 5)	36 (± 5)	*p* > 0.05
EF (%)	66 (± 5)	65 (± 6)	*p* > 0.05
IVSD	*2.47z*	1.72z	*p < 0.05*
LVIDD	0.58z	0.19z	*p* > 0.05
LVPWD	*1.15z*	0.47z	*p < 0.05*
IVSS	*2.23z*	1.91z	*p* > 0.05
LVIDS	0.32z	0.05z	*p* > 0.05
LVPWS	*1.05z*	− 0.4z	*p < 0.05*

Data from 315 adolescent elite male soccer players (SP) compared to 53 matched controls (CON). Values are presented as mean, ±SD, percent, or number. Z-scores for mean values were presented; a z-score from − 2.0 to + 2.0 was determined as normal. Significant *p* values and Z-scores > 2.0 are given in italics; Student’s *t* test

*BMI* body mass index (kg/m^2^), *BSA* body surface area, *BP* blood pressure, *LVMI* left ventricular mass indexed for BSA (g/m^2^), *LVFS* left ventricular fraction of shortening, *EF* left ventricular ejection fraction, *IVSD* interventricular septal diastolic diameter, *LVIDD* left ventricular internal diastolic diameter, *LVPWD* left ventricular posterior wall diastolic diameter, *IVSS* interventricular septal systolic diameter, *LVIDS* left ventricular internal systolic diameter, *LVPWS* left ventricular posterior wall systolic diameter

## Discussion

The morphologic cardiac adaption to exercise has been well studied in different populations of athletes and depends on the type, duration, and intensity of training. [2] Morganroth et al. described LV adaptation and concluded that pure endurance training in adults is associated with eccentric LV hypertrophy, whereas pure resistance training is characterized by concentric LV hypertrophy. This form of exercise poses a cardiac pressure load and leads to increase in left ventricular wall thickness without change in chamber size. [3]

The main findings from this study were that Z-score values for LV wall thicknesses and indexed LVM were significantly higher in SP (*p* < 0.05) suggesting cardiac adaption characteristic for resistance sports. This is in contrast with the traditional view published by Mitchell et al. showing soccer as a low-static and high-dynamic type of sport therefore predisposing more to endurance remodeling according [4]. The myocardial remodeling of soccer combines features of endurance and strength training. But the predominant LVM increase in our SP may result from specificity of training in the adolescent SP, which differs significantly from training plans in adults.

Previous studies in pre- and adolescent elite SP showed various results. In a recent study from Calo et al. among 2261 consecutive SP aged 12.4 ± 2.6 years were evaluated. Abnormal TTE was observed in 102 athletes (4.5%), including two cases of hypertrophic cardiomyopathy, eight of mild left ventricular hypertrophy, and six of mild left ventricular dilation. [8] Zdravkovic M et al. analyzed adolescent male Serbian SP (age: 12.85 ± 0.84 years) by TTE. In contrast to our findings, the authors showed significant increase of absolute LV dimensions but no differences in LV septal and posterior wall thicknesses and LV mass. [9] On the contrary, Barczuk-Falęcka M et al. studied cardiac adaption to intense exercise by cMRI in a cohort of male Polish SP (age 10.1 ± 1.4 years). Similar to our results, they were able to demonstrate significantly increased LV wall thicknesses (IVSS, LVPWD) in SP with no changes in LV cavity size and function, but with increased LVMI. [10]

These contrasting findings may be a product of differences in training load, genetic background, imaging mode, ethnicity, or some combination of factors. Normalizing LVM for inter-individual variation in body size is a central issue in human biology. During the adolescent growth spurt, variability in body size descriptors needs to be interpreted in combination with biological maturation. [11] Therefore, interpretation of echocardiography data from adolescent athletes apparently exceeding the physiologic limits of left ventricular size may require the assessment of body composition and skeletal age. [11]

There are several limitations of the present study. First of all, we did not test interobserver variability. Secondly, the size of our control group was relatively smaller in comparison to SP. Finally, our study was based on a male Caucasian population. Therefore, our results should not be extended to the general population.

## Conclusion

Our findings suggest that German elite soccer training in adolescent male is a type of sport predominantly related to cardiac resistance remodeling. A small subgroup of SP did even develop supernormal LV wall Z-score values, but did not exceed + 2.5z. We therefore recommend to rule out a primary or secondary cardiomyopathy (e.g., by advanced echocardiography techniques, cMRI, and genetic counseling) if there is a Z-score value above + 2.5 in any LV dimension.

## Abbreviations

cMRI: Cardiac magnetic resonance imaging
BSA: Body surface area in m^2^
CON: Controls
IVSS: Interventricular septal systolic diameter
LV: Left ventricle
LVM: Left ventricular mass
LVMI: Left ventricular mass indexed for BSA (g/m^2^)
LVPWD: Left ventricular posterior wall diastolic diameter
SP: Soccer player
TTE: Transthoracic two-dimensional echocardiography
